# Dietary Fat Intake: Associations with Dietary Patterns and Postmenopausal Breast Cancer—A Case-Control Study

**DOI:** 10.3390/cancers14071724

**Published:** 2022-03-28

**Authors:** Beata Stasiewicz, Lidia Wadolowska, Maciej Biernacki, Malgorzata Anna Slowinska, Ewa Stachowska

**Affiliations:** 1 Department of Human Nutrition, The Faculty of Food Science, University of Warmia and Mazury in Olsztyn, Sloneczna 45f, 10-718 Olsztyn, Poland; lidia.wadolowska@uwm.edu.pl (L.W.); malgorzata.slowinska@uwm.edu.pl (M.A.S.); 2 Department of Surgery, University of Warmia and Mazury in Olsztyn, 11-041 Olsztyn, Poland; maciej.biernacki@uwm.edu.pl; 3 Department of Human Nutrition and Metabolomics, Pomeranian Medical University, 71-460 Szczecin, Poland

**Keywords:** breast cancer, fat intake, dietary pattern, Mediterranean diet, women

## Abstract

**Simple Summary:**

Breast cancer (BC) is the most common cancer in females worldwide. Although fat has been hypothesized to be involved in BC etiology, the results of available studies are inconclusive. The aim of this study was to assess the associations of dietary fat intake, including an individual’s percentage of energy from dietary fat (Pfat) with peri- and postmenopausal breast cancer (BC) occurrence in women. The associations between Pfat and dietary patterns (DPs) were also examined. The current findings strengthen reports that a higher dietary fat intake may contribute to an increased incidence of BC, which indicates a need to reduce dietary fat intake in cancer prevention.

**Abstract:**

The aim of this study was to assess the associations of dietary fat intake with BC occurrence and dietary patterns. This case-control study involved 420 women aged 40–79 years from northeastern Poland, including 190 newly diagnosed BC cases. Dietary data were collected using a food frequency questionnaire (62-item FFQ-6^®^). The Quick Food Scan of the National Cancer Institute and the Percentage Energy from Fat Screener scoring procedures were used to estimate the percentage energy from dietary fat (Pfat). The odds of BC occurrence was three times higher in the Pfat > 32%. The Pfat > 32% was positively associated with the ‘Non-Healthy’ DP and inversely associated with the Polish-aMED^®^ score, ‘Prudent’ DP, and ‘Margarine and Sweetened Dairy’ DP. This case-control study suggests that a higher dietary fat intake (>32%) may contribute to an increased occurrence of peri- and postmenopausal breast cancer in women. Given the obtained results, an unhealthy dietary pattern characterized by the consumption of highly processed, high in sugar foods and animal fat foods should be avoided to reduce fat intake. Instead, the frequent consumption of low-processed plant foods, fish, and moderate consumption of low-fat dairy should be recommended since this pro-healthy diet is inversely associated with dietary fat intake.

## 1. Introduction

Breast cancer (BC) is the most prevalent cancer in women and the most common cause of female cancer death worldwide [1,2]. According to the GLOBOCAN statistics, breast cancer was diagnosed among over 2 million women, accounting for about 25% of all female cancers, and was a cause of 685,000 female deaths globally in 2020 [1]. Breast cancer is also the main female oncological problem in Poland, where over 18,500 women were diagnosed with this cause, contributing to 22% of all female cancers [2,3]. The risk of breast cancer increases with age [3,4]. The highest incidence of BC is observed in peri- and postmenopausal women aged over 50 years, with almost 50% of cases being diagnosed among women aged 50–69 [3]. In view of these statistics, new concepts and strategies are needed for breast cancer prevention. This task is difficult because breast cancer results from the interaction of many factors, including genetic predisposition and exposure to modifiable risk factors [4,5,6,7].

There are several modifiable and well-established causes of postmenopausal breast cancer, including alcohol drinking, lack of physical activity, and overweight or obesity throughout adulthood [4]. There is strong, convincing evidence that higher body fat (particularly visceral) and greater weight gain in adulthood can increase the risk of developing postmenopausal BC [4]. In addition to these, other factors, specifically diet and nutrients, may play a role in breast cancer etiology [4,5,6]. Regarding the macronutrients that have been hypothesized to be associated with breast cancer, fat is particularly interesting [8,9,10,11,12,13,14,15,16,17,18,19,20,21,22,23,24,25,26,27,28,29,30,31,32]. The potential mechanisms linking dietary fat with carcinogenesis are interrelated and include: an impact on oxidative stress and increased levels of the reactive oxygen species leading to DNA damage and genomic instability, modulating the expression of genes involved in cell signaling pathways, oncogenes, cell growth, differentiation, proliferation, and apoptosis [8]. Dietary fat may influence the inflammatory status through eicosanoid synthesis [8]. Fat intake may stimulate endogenous estrogen synthesis, contributing to hormonal imbalance, which is hypothesized to increase the risk of hormone-related BC [9,10]. Furthermore, a high-fat diet promotes excess body adiposity, which can lead to cancer development by the mechanisms involved in elevated insulin and insulin-like growth factor 1 (IGF-1) secretion [8].

Due to their heterogeneous nature, dietary fats may implicate cancer development, either potentially positively or negatively. This depends on many factors, including fat quality and quantity in diet. For example, intake of fish oil with a high content of n-3 polyunsaturated fatty acids (PUFAs), as well as a higher intake ratio of n-3/n-6 PUFAs, and a higher intake ratio of unsaturated to saturated fatty acids (SFAs) may play a protective, anti-cancer role whereas a high intake of animal fat (other than fish) and trans-fatty acids (TFAs) are considered to be pro-cancer [21]. The association of dietary fat intake with breast cancer risk, mortality, and survival has been discussed in numerous studies [8,9,10,11,12,13,14,15,16,17,18,19,20,21,22,23,24,25,26,27,28,29,30,31,32]. Some studies suggest a link between high fat intake and increased risk of breast cancer, whereas most of the prospective cohort studies did not find such a statistically significant association [17,24]. This inconsistency between studies could be the result of differences in study designs and sample selection and characteristics, and numerous sources of bias, regarding sample sizes, selection bias, and dietary intake measurement error [10,11,12,13,14,15,16,17,18,19,20,21,22,23,24,25,26,27,28,29,30,31,32]. In addition, the time period of dietary data collection to diagnosis and disease stage are important issues that may account for the variation among study results [10,11,12,13,14,15,16,17,18,19,20,21,22,23,24,25,26,27,28,29,30,31,32]. Based on the recent World Cancer Research Fund/American Institute for Cancer Research (WCRF/AICR) report, the evidence of associations of fat, oil, total fat, vegetable fat, and specific fatty acid, or cholesterol intake with the risk of breast cancer, both in pre- and postmenopausal women is limited and inconclusive [4]. There is also limited but generally consistent evidence suggesting that consuming a diet higher in total fat, particularly saturated fatty acids, before a diagnosis of primary breast cancer increases the risk of all-cause mortality [33].

It is important to note that people do not consume single foods or nutrients. Diet is a complex matrix of a variety of foods composed of many interrelated nutrients. An integrative approach to diet assessment as a whole is focused on dietary patterns (DPs) [7,34,35]. Thus, an assessment including the intake of nutrients such as fat (in conjunction with the derived DPs) may provide a more comprehensive view of diet in relation to the health outcomes and disease risk [34]. Available studies have shown a positive association between BC risk and Westernized DPs characterized by high consumption of animal fat, red and processed meat [36,37,38,39,40,41,42,43,44,45], and inverse associations between BC risk and a Mediterranean dietary pattern composed of fish, olive oil, vegetables, legumes, nuts and seeds, and fruit [46,47,48,49,50,51,52,53]. Besides research on associations of dietary patterns or dietary fat intake with breast cancer risk, there is a need to explore the link between dietary patterns and fat intake expressed as a component of DPs. Establishing these associations is of particular importance for determining which DPs are the main sources of fat in a given population. Nevertheless, studies in this area are limited [54].

The World Health Organization (WHO) recommends less than 30% of total energy intake from fats for the prevention of unhealthy weight gain and chronic diseases to promote successful health maintenance in adults [55]. Based on the opinions of the scientific societies and the European Food Safety Authority (EFSA) and Food and Agriculture Organization of the United Nations/World Health Organization (FAO/WHO) experts, for many populations, including American and Polish adults, there is a recommendation to limit total fat intake to 20–35% of total energy intake [56]. Instead, the average total fat intake exceeded the recommendations and was 35.1% and 37.5% among Polish women and men, and ranged from 34% to 37% of total diet energy among Americans [27,57]. Dietary fat intake among postmenopausal women is higher than 30% of total energy intake, and such a level is also maintained in breast cancer survivors [27,29]. Although the WCRF/AICR recommends limiting the consumption of fast foods and other high-fat foods, there is a lack of specific guidelines regarding the energy from dietary fat in cancer prevention [4].

Considering the above, it seems reasonable to evaluate the associations of dietary fat intake expressed in the total fat intake (Tfat), regular fat intake (Rfat), and an individual’s percentage energy from dietary fat (Pfat) with peri- and postmenopausal breast cancer occurrence in women. The associations between Pfat and DPs were also examined.

## 2. Materials and Methods

### 2.1. Study Design and Sample Collection

**Figure 1:**
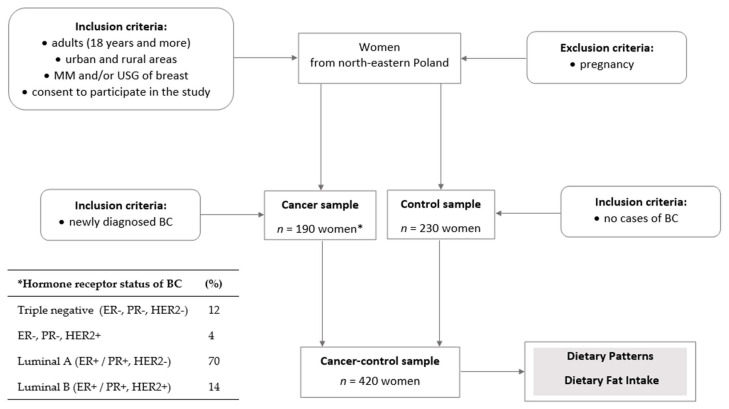
Study design and sample collection. BC—breast cancer; MM—mammography; USG—ultrasonography; * hormone receptor status of BC: ER—estrogen receptor status of tumor, PR—progesterone receptor status of tumor, HER2—human epidermal growth factor receptor 2; %—sample percentage; the stage of the study is shaded.

A case-control study was conducted in 2014–2017 among women from northeastern Poland. The cancer-control sample involved 420 subjects, aged 40.0–79.9 (mean 59.9) years, including non-randomly recruited 190 newly diagnosed and histologically confirmed breast cancer (BC) cases (cancer sample) and 230 women without breast cancer or any breast pathology based on the mammography (MM) and/or breast ultrasonography (USG) screening (control sample). A detailed flow chart of sample collection, including recruited, initial, and the final case and control samples, was shown previously [58,59]. The general inclusion and exclusion criteria of cancer and control sample collection are presented below in Figure 1. All BC cases were patients of the surgical oncology ward at the Warmia-Masuria Cancer Centre of the Ministry of the Interior and Administration Hospital, Olsztyn, Poland. The time from BC diagnosis to recruitment in the study and data collection did not exceed one month for the cancer sample.

Regarding the molecular hormone receptor status of the BC subtype, most of the cases were tumors with positive estrogen (ER+) and progesterone receptor status (PR+) and negative human epidermal growth factor receptor 2 (HER2−, 70.0%; Luminal A; Figure 1). The study excluded secondary breast cases, benign lesions, and cases after the implemented treatment or surgical intervention. The control sample was women who attended the BC screening program at medical centers in northeastern Poland. The time from BC exclusion to recruitment in the study and data collection did not exceed six months for the control sample. Detailed characteristics of the total sample are shown in Table 1 and described in the Results section.

### 2.2. Dietary Patterns Identification

Dietary data were collected using a validated non-quantitative food frequency questionnaire (62-item FFQ-6^®^), which consisted of questions about the frequency of the usual consumption of 62 food items at least 12 months prior to participation in the study [60,61]. The validation procedure of the 62-item FFQ-6^®^ was described by Niedzwiedzka et al. [60]. In brief, the test-retest reproducibility of the 62-item FFQ-6^®^ and its ability to identify dietary patterns was evaluated among young women from the Warmia and Mazury region of Poland. The test-retest reproducibility of the 62-item FFQ-6^®^ was acceptable-to-good for dietary pattern identification and good or very good for most food items, with a tendency to be higher in older age groups of females. The higher predicted repeatability of the FFQ could result from the greater precision of responses in adults than in younger individuals, which was also confirmed in the wide application of the 62-item FFQ-6^®^, e.g., in a study of a genetic-specific nutritional intervention involving adult patients with non-alcoholic fatty liver disease [62].

The 62-item FFQ-6^®^ contains “the check-up questions” which were used to verify the answers given by the respondent concerning the usual frequency of consumption of selected food items, which are usually overestimated (e.g., fruits, vegetables) or underestimated (e.g., sweets and snacks), and then to identify reliable and unreliable respondents and select a data set for the final analysis [60]. In the present study, the FFQ was administered by well-trained interviewers in face-to-face interviews, which increases the reliability of the obtained dietary data compared to the self-administered FFQ [63]. During the interview, the interviewer had the opportunity to observe the behavior of the respondent and evaluate his involvement in answering the questions [63].

The frequency of consumption expressed in six categories that ranged from ‘never or almost never’ to ‘several times a day’ was converted to the frequency of consumption per day from ‘0 time/day’ to ‘2 times/day’. Next, the consumption frequency of some food items was aggregated into 21 food groups and was then standardized and input into the principal component analysis (PCA) to dietary patterns identification. This *a posteriori* approach allows presenting the consumption frequency of many food items, and it uncovers the associations between them into a smaller set of variables in several dietary patterns [64]. Identifying DPs has been widely used in various subpopulations across the world to describe diet as a whole and find associations between diet and health outcomes [32,34,35,36,39,40,41,42,43,44,45,46,65]. PCA is the most widely used statistical technique for the identified DPs [64]. In selecting the number of PCA-derived DPs, three criteria were considered: (i) the eigenvalues from the correlation matrix of the standardized variables > 1.0, (ii) the break point identified in the plot of eigenvalues, and (iii) the total variance explained. Dietary patterns were called according to the food groups with the highest values of the factor loadings within each of the DPs [64].

The PCA-derived DPs have been described previously [58]. Briefly, the ‘Non-Healthy’ DP was characterized by the relatively frequent consumption of refined cereals, red/processed meats, sugar/honey/sweets, potatoes, animal fats, vegetable oils, and sweetened beverages/energy drinks. The ‘Prudent’ DP was characterized by the consumption of fruit, fish, legumes, milk/fermented milk drinks/cheese curd, wholemeal cereals, fruit/vegetable/vegetable-fruit juices, eggs, vegetables, nuts/seeds, vegetable oils, breakfast cereals, and cheese. The consumption of margarine/mayonnaise/dressings, sweetened milk beverages/flavored cheese curds, white meat, and breakfast cereals were characteristic for the third DP labeled as ‘Margarine and Sweetened Dairy’ (Table S1). For each DP, the scores expressed in points, as well as tertile intervals, were calculated. The consumption frequency of 21 food groups by tertiles of DPs is shown in Supplementary Table S2.

The Mediterranean diet score in the Polish adaptation (Polish-aMED^®^) was calculated based on a priori dietary assessments and a literature review. This score was chosen because of (i) the revealed many health benefits of the Mediterranean diet in a reduction in the number of non-communicable diseases, including cardiovascular disease and cancer, and (ii) for an assessment of the dietary sources of fats in the present study sample [32,47,48,49,50,51,52,53]. The full description of the Polish-aMED^®^ score development was presented previously [59]. In brief, the input variables of the Polish-aMED^®^ score were the qualitative data of the consumption frequencies (times/day) of seven selected food items: vegetables, fruit, whole grains, fish, legumes, nuts/seeds, and red/processed meats, as well as the ratio of the consumption frequency of vegetable oils to animal fat. The Polish-aMED^®^ score was calculated based on the sum of points (1 or 0) assigned the frequency of consumption of eight components related to the medians data for the initial control sample (Table S3). The Polish-aMED^®^ score was expressed in a range from 0 to 8 points, and it was considered at two levels established a priori: lower (0–4 points) and higher (5–8 points). A higher score of the Polish-aMED^®^ indicates a higher adherence to the Mediterranean diet. The characteristics of the Polish-aMED^®^ score, including correlation with food items, are shown in Supplementary Tables S1 and S2.

### 2.3. Dietary Fat Intake Assessment

In the study, the Quick Food Scan of the National Cancer Institute (USA) and the Percentage Energy from Fat Screener scoring procedures were used to estimate an individual’s percentage energy from dietary fat (Pfat) [66]. All subjects were asked about the frequency of the usual consumption of fifteen food items over the past twelve months. The consumption frequency was converted (eight categories to choose from) into the number of times consumed per day as follows: ‘never’ = 0; ‘less than once a month’ = 0.018; ‘1–3 times per month’ = 0.066; ‘1–2 times per week’ = 0.214; ‘3–4 times per week’ = 0.499; ‘5–6 times per week’ = 0.784; ‘1 time per day’ = 1; ‘2 or more times per day’ = 2. For each subject, the median values of the age- and sex-specific portion sizes for each food item were multiplied by the consumption frequency expressed in times/day.

The sum of the frequencies of the margarine, butter, or oil addition to food (on bread, rolls, pancakes; vegetables including potatoes; and rice or pasta) was calculated as total fat (Tfat). Next, the regular fat (Rfat) was estimated as a result of the multiplication of the Tfat and the equivalents related to the question about the use of low-fat margarine, as follows: ‘didn’t use or almost never’ = Tfat ∗ 1; ‘about 1/4 of the time’ = Tfat ∗ 0.75; ‘about 1/2 of the time’ = Tfat ∗ 0.50; ‘about 3/4 of the time’ = Tfat ∗ 0.25; ‘almost always or always’ = Tfat ∗ 0.

An individual’s percentage of energy from dietary fat was estimated by applying sex-specific regression coefficients for adults 18 and over to each of twelve food items (b1–b12) and Rfat (b13) in the following equation:

Percentage of energy from dietary fat = intercept + (b1 ∗ cereal) + (b2 ∗ skim milk) + (b3 ∗ eggs) + (b4 ∗ bacon) + (b5 ∗ citrus juice) + (b6 ∗ fruit) + (b7 ∗ hot dogs) + (b8 ∗ cheese) + (b9 ∗ fries) + (b10 ∗ mayonnaise) +  (b11 ∗ salad dressings) + (b12 ∗ rice) + (b13 ∗ regular fat).
(1)

In the Percentage of Energy from Fat Screener scoring procedures, the data source for portion sizes and regression coefficients was the U.S. Department of Agriculture’s 1994–1996 Continuing Survey of Food Intakes by Individuals (CSFII) [67]. Although survey data were collected in 1998, the CSFII is still one of the major large dietary intake surveys conducted in the United States involving quantitative assessment methods to derive the main dietary fat sources in adults [67].

### 2.4. Statistical Analysis

The continuous variables (e.g., consumption frequency of single food items expressed in times/day or gram/day, Tfat and Rfat scores expressed in gram/day, Pfat score expressed as a percentage, DPs scores expressed in points) were presented as means and standard deviations (SDs). For these variables, the differences between groups were verified with a Kruskal–Wallis test [64]. The Tfat, Rfat, and Pfat were also categorized into tertiles (bottom, middle, upper) based on the distribution of the total sample to divide subjects into three categories. The categorical variables were shown in sample percentages. The percentage distributions of Tfat, Rfat, and Pfat were compared between cancer cases and controls, and the percentage distributions of DPs were compared by tertiles of Pfat using the Pearson chi^2^ test with Yates’ correction as necessary [64].

A logistic regression analysis was performed to estimate the odds ratio (OR) and 95% confidence interval (95% CI) of the breast cancer occurrence in association with the adherence to the Tfat, Rfat, and Pfat, as well as to estimate the OR of the Pfat in association with the adherence to DPs. The reference categories (OR = 1.00) were the control sample and the bottom tertiles of Tfat, Rfat, Pfat, or each DP. The ORs of BC for a one-gram increase in Tfat/Rfat score, and one percent of Pfat score, and the ORs of the Pfat for a one-point increase in each DP score were also calculated. Two models were created: the crude model and the model adjusted for the potential confounders. In the ORs of BC occurrence assessment, the set of confounders included: age, BMI, socioeconomic status, overall physical activity, smoking status, abuse of alcohol, menopausal status, age at menarche, number of full-term pregnancies, oral contraceptive use, hormone-replacement therapy use, family history of breast cancer in the first- or second-degree relatives, vitamin/mineral supplements use, and breast cancer molecular subtypes. In the ORs of the Pfat assessment, the set of confounders included: age, BC cases, BMI, and socioeconomic status. The confounders were described in Supplementary Table S4. The level of significance of OR was verified with Wald’s test [64]. Statistical analyses were performed using the STATISTICA software (version 13.0 PL; StatSoft Inc., Tulsa, OK, USA; StatSoft, Krakow, Poland). The level of statistical significance was defined at a *p*-value < 0.05.

## 3. Results

The baseline sample characteristics by the percentage of energy from dietary fat are shown in Table 1. Most women were postmenopausal (85.2%). In the upper tertile of the Pfat (>32% vs. <30%), the number of breast cancer cases was approximately 21% points (55.7% vs. 34.5%) higher than in the bottom tertile. When compared to the women with the Pfat, which did not exceed 32%, the women with the Pfat above 32% were slightly younger, had a lower socioeconomic status, including lower education level. Regarding lifestyle data, more women with Pfat above 32% were smokers and declared high physical activity at work. There were fewer women with Pfat above 32% who declared a family history of breast cancer in a first- or second-degree relative or whoever used hormone-replacement therapy. The overall physical activity or BMI did not differentiate the values of Pfat (Table 1).

### 3.1. Dietary Fat Intake and Dietary Patterns

**Figure 2:**
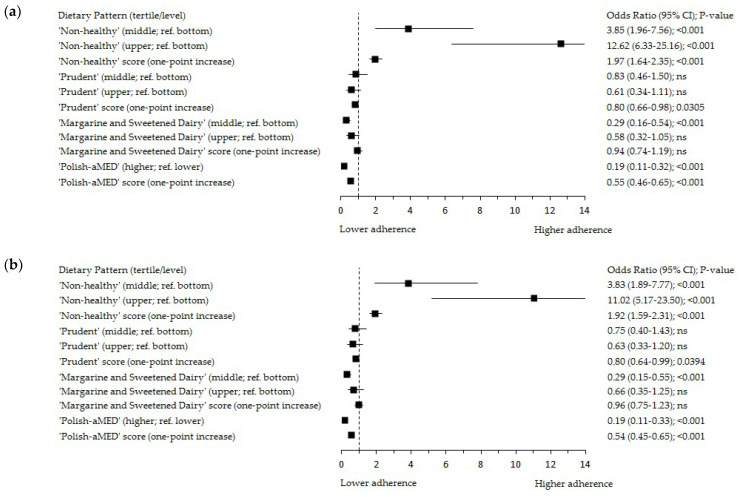
Forest plots of the upper tertile of the percentage of energy from dietary fat (>32%) by adherence to the dietary patterns among peri- and postmenopausal women (*n* = 420): (**a**) crude model; (**b**) model adjusted for: age (years), breast cancer cases, BMI (kg/m^2^), and socioeconomic status (low, average, high). Polish-aMED^®^—‘Polish-adapted Mediterranean Diet’ (range of points: 0–8); Ref.—referent, the reference categories were the bottom tertile of the percentage of energy from dietary fat, and the bottom tertiles of dietary patterns; 95% CI—95% confidence interval; *p*-value—the level of significance was assessed by Wald’s test.

The results of the significant positive associations of Pfat with the ‘Non-healthy’ DP expressed in the score and tertiles, and the inverse associations of Pfat with the Polish-aMED^®^, ‘Prudent’, and ‘Margarine and Sweetened Dairy’ DPs expressed in the score were presented in Supplementary Table S5. These baseline results were confirmed in a logistic regression analysis (Figure 2). The characteristics of the single food items consumption in the Pfat tertiles are shown in Supplementary Table S6.

The odds of Pfat in the upper tertile (>32%) were almost four times (OR: 3.83; 95% Cl: 1.89–7.77; *p* < 0.001; adjusted model; OR: 3.85; 95% Cl: 1.96–7.56; *p* < 0.001; crude model; reference: bottom tertile), and more than eleven times (OR: 11.02; 95% Cl: 5.17–23.50; *p* < 0.001; adjusted model; OR: 12.62; 95% Cl: 6.33–25.16; *p* < 0.001; crude model; reference: bottom tertile) higher in the middle and upper tertile of the ‘Non-Healthy’ DP, respectively (Figure 2a,b). A one-point increase in the ‘Non-Healthy’ DP increased the odds of Pfat > 32% almost twice (OR: 1.92; 95% Cl: 1.59–2.31; *p* < 0.001; adjusted model; OR: 1.97; 95% Cl: 1.64–2.35; *p* < 0.001; crude model).

The odds of Pfat > 32% were 81% (OR: 0.19; 95% Cl: 0.11–0.33; *p* < 0.001; adjusted model; reference: lower level 0–4 points; Figure 2b) lower in the higher level of the Polish-aMED^®^ score (5–8 points). A one-point increase in the Polish-aMED^®^ score decreased the odds of Pfat > 32% by 46% (OR: 0.54; 95% Cl: 0.45–0.65; *p* < 0.001; adjusted model). A one-point increase in the ‘Prudent’ DP decreased the odds of Pfat > 32% by 20% (OR: 0.80; 95% Cl: 0.64–0.99; *p* < 0.05; adjusted model). In the middle tertile of the ‘Margarine and Sweetened Dairy’ DP, the odds of Pfat > 32% was 71% lower (OR: 0.29; 95% Cl: 0.15–0.55; *p* < 0.001; adjusted model; reference: bottom tertile; Figure 2b). These associations support the results from the crude models (Figure 2a).

### 3.2. Dietary Fat Intake and Breast Cancer

**Figure 3:**
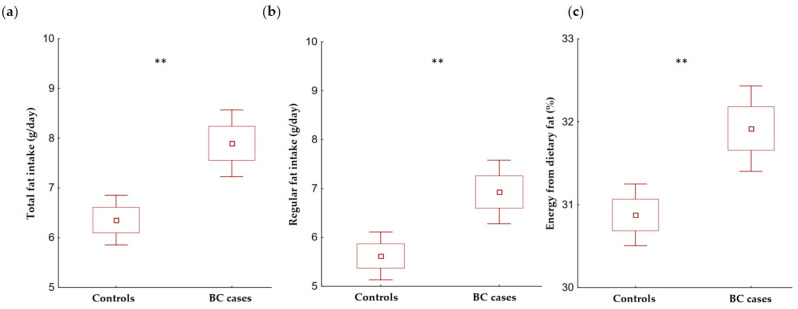
The mean and standard deviation (SD) of total fat intake (**a**), regular fat intake (**b**), and the percentage of energy from dietary fat (**c**), among breast cancer cases (*n* = 190) and controls (*n* = 230). BC—breast cancer; the procedure of the total fat intake, regular fat intake, and percentage of energy from the dietary fat calculation is given in the Materials and Methods section; *p*-value—the level of significance was assessed by Kruskal–Wallis test; ** *p* < 0.01.

Breast cancer cases had, on average, a higher total fat intake (7.9 vs. 6.4 g/day) as well as regular fat intake (6.9 vs. 5.6 g/day) and the percentage of energy from dietary fat (31.9% vs. 30.9%) compared with the controls (Figure 3a–c).

**Figure 4:**
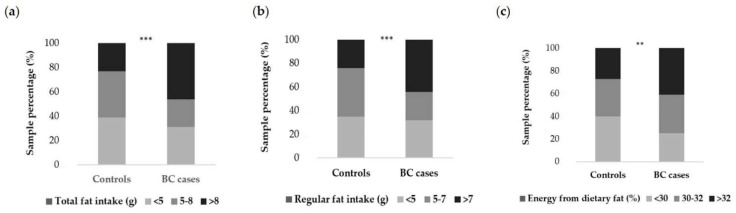
Total fat intake (**a**), regular fat intake (**b**), and the percentage of energy from dietary fat (**c**) in association with breast cancer. BC—breast cancer; the procedure of the total fat intake, regular fat intake, and percentage of energy from dietary fat calculation is given in the Materials and Methods section; *p*-value—level of significance assessed by chi^2^ test; ** *p* < 0.01, *** *p* < 0.001.

Figure 4 presents the distribution of dietary fat intake by case-control status. Compared to the controls, more cases of breast cancer had higher levels of total fat intake above 8 g/day (46.3% vs. 23.0%), regular fat intake above 7 g/day (43.7% vs. 24.3%), and the percentage of energy from dietary fat above 32% (41.1% vs. 27.0%). The consumption of single food items components of Pfat among cancer and control samples are shown in Supplementary Table S7.

**Figure 5:**
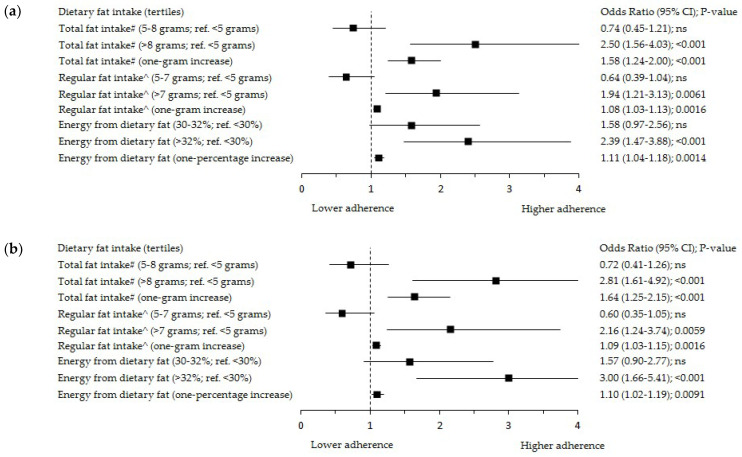
Forest plots of breast cancer occurrence by adherence to the dietary fat intake among peri- and postmenopausal women (*n* = 420): (**a**) crude model; (**b**) model adjusted for: age (years), BMI (kg/m^2^), socioeconomic status (low, average, high), overall physical activity (low, moderate, high), smoking status (non-smoker, smoker), abuse of alcohol (no, yes), menopausal status (peri-, postmenopausal), age at menarche (<12, 12–14.9, ≥15 years), number of full-term pregnancies (0, 1–2, ≥3), oral contraceptive use (no, yes), hormone-replacement therapy use (no, yes), family history of breast cancer in the first- or second-degree relatives (no, I don’t know, yes), vitamin/mineral supplements use (no, yes) and molecular of breast cancer subtypes (triple negative, ER-, PR-, HER2+ subtype, luminal A, luminal B). Ref.—referent, the reference categories were the control sample and the bottom tertiles of total fat intake, regular fat intake or percentage of energy from dietary fat; ^#^ understood as intake of total fat added to food; ^^^ understood as intake of regular fat added to food after the low-fat margarine was included; the procedure of the total fat intake, regular fat intake and percentage of energy from dietary fat calculation is given in the Section 2; 95% CI—95% confidence interval; *p*-value—the level of significance was assessed by Wald’s test.

The odds of breast cancer occurrence were almost three times higher in the upper tertile of Tfat > 8 g/day (OR: 2.50; 95% Cl: 1.56–4.03; *p* < 0.001; crude model; OR: 2.81; 95% Cl: 1.61–4.92; *p* < 0.001; adjusted model; reference: bottom tertile < 5 g/day). A one-point increase in Tfat increased the BC occurrence by 58% (OR: 1.58; 95% Cl: 1.24–2.00; *p* < 0.001) in crude model and 64% (OR: 1.64; 95% Cl: 1.25–2.15; *p* < 0.001; Figure 5a,b) in an adjusted model.

The odds of breast cancer occurrence were two-fold higher in the upper tertile of Rfat >7 g/day (OR: 1.94; 95% Cl: 1.21–3.13; *p* < 0.01; crude model; OR: 2.16; 95% Cl: 1.24–3.74; *p* < 0.01; adjusted model; reference: bottom tertile < 5 g/day). A one-point increase in Rfat intake increased the occurrence of BC by 8% (OR: 1.08; 95% Cl: 1.03–1.13; *p* < 0.01) in a crude model and 9% (OR: 1.09; 95% Cl: 1.03–1.15; *p* < 0.01) in an adjusted model.

The odds of BC occurrence were two- and three-fold higher in the upper tertile of Pfat > 32% (OR: 2.39; 95% Cl: 1.47–3.88; *p* < 0.001; crude model; OR: 3.00; 95% Cl: 1.66–5.41; *p* < 0.001; adjusted model; reference: bottom tertile < 30%). A one-point increase in Pfat increased the BC occurrence by 10% (OR: 1.10; 95% Cl: 1.02–1.19; *p* < 0.01) in an adjusted model and 11% (OR: 1.11; 95% Cl: 1.04–1.18; *p* < 0.01) in a crude model. There were no significant associations of middle tertiles of the dietary fat intake with the breast cancer occurrence (Figure 5a,b).

## 4. Discussion

To the authors’ best knowledge, this was the first study that comprehensively examined the associations of dietary fat intake assessed by the Quick Food Scan of the National Cancer Institute, U.S., with dietary patterns and breast cancer occurrence. The findings reported in the current paper strengthen those reports that have shown a positive association between higher dietary fat intake and increased incidence of breast cancer in peri- and postmenopausal women. As regards the dietary patterns, a higher adherence to the ‘Non-Healthy’ pattern contributed to obtaining the higher percentage of energy from dietary fat. A lower percentage of energy from dietary fat was associated with the higher adherence to the Polish-aMED^®^ score, and middle adherence to the ‘Margarine and Sweetened Dairy’ pattern, as well as adherence to the ‘Prudent’ pattern expressed by only a one-point increase.

### 4.1. Dietary Fat Intake in Total Sample

For the entire sample, the average dietary fat intake constituted 31.3% of total energy, which exceeds the WHO recommendation to limit the energy intake from fats to less than 30% in the prevention of chronic diseases in adults [55]. Nonetheless, the average dietary fat intake ranged from 20% to 35% of the recommended energy intake according to the EFSA [56]. The average energy from fats among Polish women in the current study was comparable with national data for U.S. women of a similar age in the Women’s Health Initiative Dietary Modification (WHI DM) trial (32.0%) [29]. Surprisingly, the current results are not consistent with findings in the general population, where the average total fat intake was 35.1% of energy among Polish women, which is greater than calculated for participants of this study [57]. However, these data were obtained from the National Multicenter Health Survey (WOBASZ II), which involved a random sample of the whole Polish female population aged at least 20 years [57]. A greater baseline percent of calories from fat than in the current sample was also reported in a WHI DM trial involving 48,835 U.S. postmenopausal women (37.6% vs. 31.3%) [24]. In turn, a lower average percentage of total energy intake from fat compared to this study was reported in a large Mexican-American BetaGene cohort (28.6% vs. 31.3%) [54]. These discrepancies could result from country-to-country differences in dietary fat intake and methods that were used to estimate dietary fat intake (FFQ vs. food records vs. 24 h dietary recall) [10,16].

### 4.2. Dietary Fat Intake and Breast Cancer

The average percentage of energy from the dietary fat intake among Polish BC cases was in accordance with the findings for the British BC cases (31.9% and 31.6%, respectively) [10]. However, in contrast to the findings of the current study, Key et al. [10] did not observe a significant difference in the percentage of energy from total fat intake between cases and controls. The current study revealed that dietary fat intake above 32% of total energy increased the occurrence of breast cancer approximately two-fold compared to the percentage of energy from dietary fat below 30%. This association increased three-fold and remained statistically significant after adjusting for many known and potential factors of BC etiology. Interestingly, there were no significant associations of the middle tertiles of the dietary fat intake (30–32%) with the breast cancer occurrence. Thus, these results suggest that dietary fat may be a strong predictor of BC when consumed in greater amounts (>32% of total energy). Similarly, results of observational studies have reported that dietary fat, and in particular animal fat, may be associated with a greater risk of BC. For example, in another Polish case-control study conducted in the Region of Western Pomerania, the consumption of 5–6 servings of animal fat per week significantly increased OR of BC from 1.7 to 2.9 times, compared with the consumption of 2 or fewer servings of animal fat per week [12]. Dietary fat intake could affect the progression of breast cancer through several potential mechanisms [5,6,9,14,54]. Current evidence indicates that a higher fat intake is associated with obesity [4]. There is strong evidence that obesity (particularly greater body fatness in adulthood) is a convincing cause of postmenopausal breast cancer [4]. After menopause, visceral adipose tissue is a major source of endogenous estrogens [4]. Adiposity can increase de novo estrogen synthesis leading to increased cell proliferation and breast cancer [9]. To assess fat distribution in adults, BMI is interpreted together with the waist circumference and the waist-to-height ratio [4]. Surprisingly, in the current study, none of the discussed anthropometric parameters differed in dietary fat intake. These results indicate that the anthropometric measures are imperfect and cannot reliably distinguish between total and abdominal fat or between visceral and subcutaneous fat [4]. Another explanation is the hypothesis that animal fat intake increases the synthesis of estrogens from androstenedione and insulin levels, i.e., compounds that promote tumor growth [14,54]. A positive link between fat intake and estradiol levels has been noted in the present study, in which 84% of cases were estrogen-related BC, and in the prospective WHI DM study, which found a statistically significant reduction in BC risk after the implementation of low-fat DP only for estrogen receptor (ER)-positive and progesterone receptor (PR)-negative tumors (HR, from 0.64 through the 0.70 to 0.77) [17,18,24,29].

According to the WCRF/AICR report, there is limited but generally consistent evidence suggesting a positive association between the intake of total fat, particularly saturated fatty acids, before a diagnosis of primary breast cancer and the risk of all-cause mortality [33]. This association was supported in the last WHI DM study, in which beneficial long-term effects of low-fat DP in reducing the incidence of deaths as a result of BC (HR, 0.79), as well as a result of any cause (HR, from 0.65 to 0.84), were observed, with a notional threshold of 28% calories from fat [17,18,30]. However, it has been supposed that these positive findings could result from the beneficial effect of a low-fat diet on reduced mortality of other causes, including cardiovascular disease [18,30]. Similarly, as in the current study, a positive association between total fat intake and BC has also been found among premenopausal women in the Nurses’ Health Study II (NHSII); however, this negative effect has been observed at the higher level of fat intake (>35% energy vs. ≤25% energy) [23]. In premenopausal women, besides the estrogen influence, the developing breast tissue is more sensitive to structural changes than in the postmenopausal period [27]. Therefore, it is biologically plausible that a high-fat diet can increase breast density as a strong predictor of breast cancer [14,25,27].

Although some studies have suggested a detrimental effect of total dietary fat intake on breast cancer, most results were not statistically significant. There were no reported significant associations of dietary total fat intake and specific types of fat with BC risk among postmenopausal women in many large prospective cohort studies within the NHSII, the U.K. Dietary Cohort Consortium, and meta-analysis [10,16,22,23]. These findings were obtained irrespective of whether a diet was estimated by food records or FFQs as in the current study. A dietary recommendation to reduce fat for cancer prevention after menopause was generally also not supported by the intervention WHI DM study, where the reduction in dietary fat by 8–10% of total energy did not show a significant decrease in BC occurrence [24,29]. However, contrary to the present study, there was no evidence of a difference in total fat intake between the intervention and control groups at the end of the study, which could explain the null result [29]. Moreover, women who reported a relatively higher percentage of energy from dietary fat at baseline (≥36.8% of energy from fat) made larger reductions in fat intake and demonstrated a nominally significant reduction in breast cancer risk (HR 0.76) [24]. Speculatively, this may suggest that any protective effect of a low-fat intervention does not carry forward after stopping the intervention when dietary fat intake tends to increase.

### 4.3. Dietary Fat Intake and Dietary Patterns in Associations with Breast Cancer

Due to the occurrence of the potential association between dietary fat intake and breast cancer, it is important to characterize DPs as the sources of dietary fat to understand how dietary patterns affect dietary fat levels. In the present study, the percentage of energy from dietary fat intake (>32%) was strongly and positively associated with the adherence to the ‘Non-Healthy’ DP. Since there is a lack of similar studies in this area, a comparison with other results is difficult. Hence, this study indicates a large knowledge gap that remains to be addressed to better understand these associations with breast cancer. The new data from the present analysis, along with the previously reported data [58], provide evidence of the strong harmful effect of the pattern known as ‘Non-Healthy’. This pattern increased the risk of breast cancer approximately three-fold [58]. Thus, the supposedly ‘Non-healthy’ pattern was a major source of fat that contributed to breast cancer. This positive association is not surprising, as animal fat and processed meats have a high loading in the Polish dietary pattern [58]. However, caution is appropriate when interpreting the association between fat and breast cancer because this association could also result from other specific dietary components [23]. Besides red and processed meats and animal fat, the ‘Non-Healthy’ DP was also characterized by the frequent consumption of sweets, refined cereals, sweetened beverages, and energy drinks high in sugar [58]. These results agree with the WCRF/AICR recommendation that emphasizes the importance of limiting the consumption of sweets and processed and red meat [4]. Existing evidence indicated that these foods containing high-glycemic carbohydrates and saturated and trans-fatty acids can lead to cancer development and tumor growth by the mechanisms involved in elevated insulin and IGF-1 secretion and proinflammatory status [8,19,21].

Since both the quantity and quality of dietary fat intake are important in the context of BC incidence, the analyses with Pfat also involved the Mediterranean diet (MED) score. This may be particularly crucial, considering that in several Mediterranean countries, the percentage of energy from dietary fat can easily exceed 30%, although it is mainly of mono- and polyunsaturated origin and is still perfectly in agreement with the national dietary guidelines [32]. In general, the traditional Mediterranean diet is not a typical low-fat pattern due to the relatively high consumption of olive oil in the Mediterranean populations [47,50]. In the present analysis, the adherence to the Polish-aMED^®^ score was inversely associated with the percentage of energy from dietary fat. Similar to the traditional MED, the Polish-aMED^®^ score was also composed of olive oil and other vegetable oils; however, they are not consumed in great amounts in non-Mediterranean countries. The Polish-aMED^®^ score was also positively correlated with the consumption of nuts and seeds, wholemeal cereals, fruit, legumes, vegetables, and fish [58,59]. Thus, the Polish-aMED^®^ score represents a plant-fish-based diet as a source of MUFAs, PUFAs, and many other bioactive compounds such as antioxidants and chemopreventive factors with an anti-cancer potential [11,13].

The current findings could explain results from the previous analysis, where the higher adherence to the Polish-aMED^®^ score reduced the risk of breast or lung cancer risk [59]. There is evidence that marine n-3 PUFAs have anti-inflammatory properties [38]. Another explanation is that PUFA intake has a beneficial effect on the balance of lipid components in the breast cell membrane and its fluidity. This, in turn, could affect a cell’s response to growth factors or its ability to adhere to neighboring cells [49].

An inverse association with the percentage of energy from dietary fat was also found for the ‘Prudent’ DP among Polish postmenopausal women. A statistically significant association was observed only for a one-point increase in the pattern score, which could be due to the greater sensitivity of continuous data compared to dichotomous data [64]. Although the ‘Prudent’ DP was also characterized by the frequent consumption of plant foods and fish, similarly to the Polish-aMED^®^ score, it was also composed of the consumption of dairy and eggs. Therefore, the ‘Prudent’ DP could contribute to greater amounts of dietary fat than the Polish-aMED^®^ score. This could explain the weaker inverse association of the ‘Prudent’ DP with Pfat in comparison to the Polish-aMED^®^ score. Due to its composition, the ‘Prudent’ DP provided both PUFAs, and SFAs, which could result in the neutral character in relation to breast cancer incidence [58]. An interesting finding of the current analyses was the inverse association between the higher percentage of energy from dietary fat (>32%) and the adherence to the ‘Margarine and Sweetened Dairy’ DP, although this association was only significant for the middle adherence to this pattern. Possibly due to modest consumption of other fats (margarine, mayonnaise, dressings), sweetened milk beverages and flavored cheese curds, white meat, and breakfast cereals, the ‘Margarine and Sweetened Dairy’ pattern did not heavily contribute to Pfat increase and was not associated with the breast cancer risk [58].

### 4.4. Strengths and Limitations

The main strength of this study is a comprehensive evaluation of dietary data in the context of breast cancer. The current analyses have not been limited only to the assessment of the association between dietary fat intake and BC occurrence. The authors also examined the main dietary patterns, including the Polish-aMED^®^ score, and the link between these dietary patterns with dietary fat intake. Other strengths include the dietary assessment using two validated FFQs. FFQs are often used in dietary assessment because they are better to describe the usual diet than other methods [60,63,65]. The Quick Food Scan of the National Cancer Institute and the Percentage Energy from Fat Screener scoring procedures were used to estimate the individual’s percentage of energy from dietary fat, which was adapted from the Nurses’ Health Study (NHS) [66]. This validated tool has been used in large population studies, including the National Health Interview Survey (NHIS) Cancer Control Supplements (CCS) [66]. The Quick Food Scan of the National Cancer Institute is easy, quick to administer, and less engaging for the respondent, and, based on the consumption frequency of selected foods, it is possible to assess the total fat intake. Following the procedure achieves the same results as with the quantitative FFQ, but compared to the quantitative FFQ, it does not burden the respondents [63,66]. In the present study, the questionnaires were administered by well-trained interviewers in face-to-face interviews (which increases the reliability of the obtained dietary data compared to the self-administered FFQ) [63]. This study includes a comprehensive adjustment for many known and potential confounders related to both breast cancer and fat intake (socioeconomic, reproductive, lifestyle, anthropometric, family history of breast cancer, and breast cancer molecular subtype variables). Similar results of crude and adjusted models suggest little confounding by known covariates.

This study has some potential limitations, which also need to be considered when interpreting these results. Dietary data and fat intake were assessed using the FFQ, while some studies have shown that the FFQs tend to over-report healthy foods and under-report unhealthy food consumption [20,24,63]. However, the FFQs’ verification questions allowed reliable and unreliable respondents to be identified and to select a data set for the final analysis [60]. The use of more precise quantitative FFQ or multi-day food records to validate data collected with the FFQs would be too burdensome for the respondents, especially BC cases, and could result in discouragement or refusal of many respondents to participate in the study [63]. Nevertheless, the available evidence from validation studies suggests that both methods provide reasonably valid estimates of dietary fat intake [10,60]. Moreover, the previously reported results of a low blood concentration of vitamins and minerals and disturbed lipid profile confirm the low quality of high-fat content diet in BC cases [70]. Second, although it is known that there are some differences in food consumption among the Polish and American populations [12,29,57], it was decided to use the Quick Food Scan of the National Cancer Institute (USA) and the Percentage Energy from Fat Screener scoring procedures to estimate an individual’s percentage of energy from dietary fat in the Polish case-control study. This was a result of the similarity of the dietary fat intake of Poles to Americans and the influence of Western patterns, which has been recently observed [29]. Moreover, there was no similar short FFQ to estimate the usual dietary intake of the percentage energy from fat, adapted in Polish studies, including cancer clinical trials. In Poland, there are some semi-quantitative FFQs such as a full semi-quantitative food frequency questionnaire (165-item FFQ); however, the 165-item FFQ is difficult to apply due to a large number of detailed questions, which is inconvenient for the respondents [60]. Further, although both total fat and different kinds of dietary fat (saturated/trans vs. mono/polyunsaturated) intake are important in the context of BC incidence, these analyses were limited to examining the associations of the percentage of energy from total fat with BC occurrence. A Fat Screener was used, which provided food frequency consumption data and allowed only the total fat intake to be estimated. To assess fatty acid intake, it is necessary to use the multi-day food records in long-term prospective studies [63]. This important point will be investigated in our next study.

## 5. Conclusions

This case-control study suggests that a higher dietary fat intake (>32%) may contribute to an increased occurrence of peri- and postmenopausal breast cancer in women.

Given the obtained results, an unhealthy dietary pattern characterized by the consumption of highly processed, high in sugar foods and animal fat foods should be avoided to reduce fat intake. Instead, the frequent consumption of low-processed plant foods and fish and moderate consumption of low-fat dairy should be recommended since this pro-healthy diet was inversely associated with dietary fat intake.

The current findings strengthen reports that indicate a need to reduce dietary fat intake in cancer prevention. Nevertheless, it should be noted that not all high-fat foods contribute to breast cancer development. An example is high-fat fish, which is a component of a pro-healthy diet in the present study. These findings indicate a need to take into account both total fat intake and the intake of fatty acids when the association with cancer was examined. This requires long-term prospective studies using quantitative dietary assessment methods and confirmation that the association between fat intake and cancer is independent of other components of high-fat foods and identifying underlying mechanisms.

## Figures and Tables

**Table 1:** Baseline sample characteristics by the percentage of energy from dietary fat (% or mean (SD).

Variable	Total Sample	Energy from Dietary Fat (Tertiles)			*p*-Value
		Bottom (<30%)	Middle (30–32%)	Upper (>32%)	
Sample size (number)	420	139	141	140	
Breast cancer cases (%)	45.2	34.5	45.4	55.7	0.0018
Age (years) *^#^*	59.9 (8.6)	60.4 (8.4)	60.8 (8.2)	58.6 (9.0)	0.0437
40.0–49.9	15.5	12.9	12.1	21.4	
50.0–59.9	30.0	30.9	24.8	34.3	0.0319
60.0–69.9	42.6	41.0	51.8	35.0	
70.0–79.9	11.9	15.1	11.3	9.3	
Menopausal status (%)					
Peri-menopausal	14.8	10.1	12.8	21.4	0.0200
Postmenopausal	85.2	89.9	87.2	78.6	
BMI (kg/m^2^) ^a#^	27.9 (5.0)	27.8 (4.7)	28.2 (5.1)	27.8 (5.0)	0.7735
Underweight (<18.5)	0.7	0.7	0.7	0.7	
Normal weight (18.5–24.9)	29.2	30.9	25.0	31.7	0.7914
Overweight (25.0–29.9)	39.0	35.3	44.3	37.4	
Obesity (≥30.0)	31.1	33.1	30.0	30.2	
Waist circumference (cm) ^a#^	92.0 (13.2)	90.2 (12.0)	92.4 (12.6)	93.3 (14.6)	0.1852
Waist-to-height ratio ^a#^	0.57 (0.08)	0.56 (0.08)	0.57 (0.08)	0.58 (0.09)	0.2945
Place of residence (%)					
Village	28.1	25.9	26.2	32.1	
Town (<20,000 inhabitants)	15.2	12.2	14.2	19.3	0.0652
Town (20–100,000 inhabitants)	20.5	25.2	15.6	20.7	
City (>100,000 inhabitants)	36.2	36.7	44.0	27.9	
Education level (%)					
Primary	13.6	12.2	13.5	15.0	
Secondary	58.3	48.9	60.3	65.7	0.0084
Higher	28.1	38.8	26.2	19.3	
Economic situation (%)					
Below average	16.0	14.4	13.5	20.0	
Average	71.2	68.3	73.8	71.4	0.1595
Above average	12.9	17.3	12.8	8.6	
Situation of household (%)					
We live poorly	0.2	0.0	0.7	0.0	
We live very thriftily	16.9	15.8	14.2	20.7	
We live thriftily	56.0	59.7	56.0	52.1	0.6788
We live well	24.8	23.0	27.0	24.3	
We live very well	2.1	1.4	2.1	2.9	
Socioeconomic index (points) ^b#^	9.9 (2.1)	10.1 (2.1)	10.0 (2.2)	9.5 (2.0)	0.0081
Socioeconomic status (%) ^b^					
Low	41.0	34.5	34.0	54.3	
Average	36.7	38.8	42.6	28.6	0.0030
High	22.4	26.6	23.4	17.1	
Physical activity at work (%) ^c^					
Low	54.0	60.4	61.7	40.0	
Moderate	32.6	27.3	29.8	40.7	0.0011
High	13.3	12.2	8.5	19.3	
Physical activity in leisure time (%) ^d^					
Low	22.6	17.3	26.2	24.3	
Moderate	64.3	64.0	62.4	66.4	0.0862
High	13.1	18.7	11.3	9.3	
Overall physical activity (%) ^e^					
Low	52.9	53.2	61.0	44.3	
Moderate	44.0	43.9	36.9	51.4	0.0852
High	3.1	2.9	2.1	4.3	
Smokers (%) ^f^	53.1	46.8	51.1	61.4	0.0413
Abuse of alcohol (%) ^g^	4.0	2.2	4.3	5.7	0.3175
Age at menarche (years)					
<12	12.1	8.6	12.1	15.7	
12–14.9	63.3	64.0	65.2	60.7	0.4311
≥15	24.5	27.3	22.7	23.6	
Number of full-term pregnancies (%)					
0	12.1	12.9	13.5	10.0	
1–2	61.7	66.2	63.8	55.0	0.0691
≥3	26.2	20.9	22.7	35.0	
Oral contraceptive use (%) ^h^	20.2	25.2	19.1	16.4	0.1768
Hormone-replacement therapy use (%) ^h^	16.7	25.2	12.1	12.9	0.0044
Vitamin/mineral supplements use (%) ^i^	38.6	42.4	35.5	37.9	0.4755
Family history of BC (%) ^j^	19.3	27.3	19.1	11.4	0.0104
Diagnosed chronic diseases (%)	56.9	61.2	57.4	52.1	0.3114

BC—breast cancer; BMI—body mass index was calculated using measured weight and height; ^a^ anthropometric data were obtained for *n* = 409; ^b^ was calculated on the basis of place of residence, educational level, and declared economic situation [59]; ^c^ physical activity at work: “low”—more than 70% of working time spent sedentary or retired, “moderate”—approximately 50% of working time spent sedentary and 50% of working time spent in an active manner, “high”—approximately 70% of working time spent in an active manner or physical work related to great exertion [68]; ^d^ physical activity in leisure time: “low”—sedentary for most of the time, watching TV, reading books, walking 1–2 h per week, “moderate”—walking, bike riding, gymnastics, gardening, light physical activity performed 2–3 h per week, “high”—bike riding, jogging, gardening, sport activities involving physical exertion performed more than 3 h weekly [68]; ^e^ after combining data based on declared physical activity at work and physical activity in leisure time [69]; ^f^ current or former-smokers; ^g^ consumption of at least 1 bottle (0.5 L) of beer or 2 glasses of wine (300 mL) or 2 drinks (300 mL) or 2 glasses of vodka (60 mL) per day [4]; ^h^ ever use; ^i^ self-declared use of vitamin and/or mineral supplements within the last 12 months; ^j^ in first- or second-degree relative; %—sample percentage; ^#^ mean and standard deviation (SD); *p*-value—level of significance verified with chi^2^ test (categorical variables) or Kruskal–Wallis test (continuous variables).

## Data Availability

The data presented in this study are available on request from the corresponding author.

## Supplementary Materials

The following are available online at https://www.mdpi.com/article/10.3390/cancers14071724/s1, Table S1: Factor loadings for food groups in PCA-derived dietary patterns and the Pearson’s correlation coefficients for Polish-aMED^®^ score among peri- and postmenopausal women (*n* = 420), Table S2: The mean (95% CI) of food consumption and fat intake by dietary patterns among peri- and postmenopausal women (*n* = 420), Table S3: Description of food groups for the ‘Polish-adapted Mediterranean Diet’ score (0–8 points) calculation–data for the initial control sample (*n* = 242), Table S4: Confounders in the case-control study regarding the association of dietary fat intake with dietary patterns and breast cancer occurrence, Table S5: The dietary fat intake in association with dietary patterns (% or mean (SD)), Table S6: The consumption of single foods and the assessment of fat content in the diet by the percentage energy from dietary fat status (% or mean (SD)), Table S7: The consumption of single foods and the assessment of fat content in the diet by the by case-control status (% or mean (SD)). Reference [58] is cited in the Supplementary Materials.
