# Supplementary material for: Dietary Fat Intake: Associations with Dietary Patterns and Postmenopausal Breast Cancer—A Case-Control Study
Source: Cancers (Basel). 2022 Mar 28;14(7):1724. doi: 10.3390/cancers14071724 (PMC8997044; doi:10.3390/cancers14071724)
Supplement: Supplementary file 1 [file cancers-14-01724-s001.zip › cancers-1630961-supp-final.pdf]

Supplementary Materials

Dietary Fat Intake: Associations with Dietary Patterns and Postmenopausal Breast Cancer—A Case-Control Study

Beata Stasiewicz, Lidia Wadolowska, Maciej Biernacki, Malgorzata A. Slowinska and Ewa Stachowska

Table S1. Factor loadings for food groups in PCA-derived dietary patterns and the Pearson's correlation coefficients for 'Polish-aMED' score among peri- and post-menopausal women ($n = 420$).

Food Groups	PCA-Derived Dietary Patterns			'Polish-aMED' Score
	'Non-Healthy'	'Prudent'	'Margarine and Sweetened Dairy'	
Refined cereals	**0.67**	−0.25	0.12	−0.41 *
Red and processed meats	**0.63**	0.06	0.07	**−0.34 ***
Sugar, honey and sweets	**0.57**	0.13	0.04	−0.14 *
Potatoes	**0.55**	−0.04	−0.02	−0.20 *
Animal fats	**0.49**	0.12	**−0.66**	−0.31 *
Vegetable oils (including olive oil)	**0.34**	**0.36**	0.02	0.14 *
Sweetened beverages and energy drinks	**0.32**	0.13	0.18	0.01
Fruit	−0.06	**0.55**	−0.05	**0.38 ***
Fish	−0.07	**0.49**	0.09	**0.33 ***
Legumes	0.00	**0.48**	0.19	**0.36 ***
Milk, fermented milk drinks and cheese curd	−0.05	**0.48**	0.13	0.22 *
Wholemeal cereals	**−0.45**	**0.47**	−0.01	**0.43 ***
Fruit, vegetable, vegetable-fruit juices	0.16	**0.45**	0.00	0.11 *
Eggs	0.20	**0.44**	−0.10	0.10 *
Vegetables	0.00	**0.42**	−0.17	**0.34 ***
Nuts and seeds	**−0.39**	**0.42**	−0.12	**0.46 ***
Breakfast cereals	0.04	**0.35**	**0.31**	0.14 *
Cheese	0.23	**0.34**	0.10	0.02
Other fats (margarine, mayonnaise, dressings)	0.17	−0.12	**0.80**	−0.05
Sweetened milk beverages and flavoured cheese	0.29	0.28	**0.36**	−0.06
White meat	0.22	0.08	**0.31**	−0.03
Ratio of vegetable oils to animal fat	NA	NA	NA	**0.38 ***
Share in explaining the variance (%)	13	12	7	NA

'Polish-aMED'—'Polish-adapted Mediterranean Diet' (range of points: 0–8); PCA—Principal Component Analysis; NA—not applied; bolded values are marked for the main components of PCA-derived dietary patterns with absolute loadings ≥ 0.3 and for eight components of the 'Polish-aMED' score; *$p < 0.05$, test of significance for Pearson's correlation coefficients. In identifying the number of PCA-derived DPs, three criterions were considered: (i) the eigenvalues from the correlation matrix of the standardized variables > 1.0, (ii) the break point identified in the plot of eigenvalues, and (iii) the total variance explained.

Table S2. The mean (95% CI) of food consumption and fat intake by dietary patterns among peri- and post-menopausal women (n = 420).

Food groups	Total	Dietary Patterns										
		'Non-Healthy' tertiles			'Prudent' tertiles			'Margarine and Sweetened Dairy' tertiles			'Polish-aMED' levels	
		bottom	middle	upper	bottom	middle	upper	bottom	middle	upper	lower	higher
Sample size (n)	420	139	141	140	139	140	141	140	139	141	189	231
**Frequency of food consumption (times/day)**[#]												
Sugar, honey and sweets	1.9 (1.7; 2.0)	1.2 (1.0; 1.3)	1.7 (1.5; 1.8)	2.8 (2.6; 3.0)	1.7 (1.5; 1.9)	1.9 (1.7; 2.1)	2.1 (1.8; 2.3)	2.0 (1.7; 2.2)	1.7 (1.5; 1.9)	2.0 (1.8; 2.2)	2.0 (1.8, 2.2)	1.8 (1.6, 1.9)
Red and processed meats	1.4 (1.3; 1.4)	0.8 (0.7; 0.9)	1.4 (1.3; 1.5)	1.9 (1.8; 2.0)	1.3 (1.2; 1.4)	1.3 (1.2; 1.5)	1.5 (1.3; 1.6)	1.4 (1.3; 1.5)	1.3 (1.1; 1.4)	1.4 (1.3; 1.5)	1.6 (1.5, 1.7)	1.2 (1.1, 1.3)
Animal fats	1.1 (1.1; 1.2)	0.7 (0.6; 0.8)	1.1 (1.0; 1.2)	1.6 (1.5; 1.8)	1.0 (0.9; 1.1)	1.2 (1.1; 1.4)	1.2 (1.1; 1.4)	1.9 (1.7; 2.0)	1.0 (0.9; 1.2)	0.5 (0.5; 0.6)	1.4 (1.3, 1.5)	0.9 (0.8, 1.0)
Milk, fermented milk drinks and cheese curd	1.1 (1.0; 1.1)	1.2 (1.0; 1.3)	1.0 (0.9; 1.1)	1.1 (1.0; 1.2)	0.7 (0.6; 0.8)	1.1 (1.0; 1.2)	1.4 (1.3; 1.6)	0.9 (0.8; 1.0)	1.2 (1.1; 1.3)	1.1 (1.0; 1.2)	1.0 (0.9, 1.1)	1.2 (1.1, 1.2)
Refined cereals	0.9 (0.9; 1.0)	0.4 (0.3; 0.4)	0.9 (0.8; 1.0)	1.5 (1.4; 1.6)	1.2 (1.1; 1.3)	0.8 (0.7; 0.9)	0.8 (0.7; 0.9)	0.9 (0.8; 1.1)	0.8 (0.7; 0.9)	1.1 (1.0; 1.2)	1.2 (1.1, 1.3)	0.7 (0.6, 0.8)
Vegetables	1.2 (1.2; 1.3)	1.2 (1.1; 1.3)	1.2 (1.2; 1.3)	1.2 (1.1; 1.3)	1.0 (0.9; 1.1)	1.2 (1.1; 1.3)	1.5 (1.4; 1.6)	1.3 (1.3; 1.4)	1.2 (1.1; 1.3)	1.1 (1.0; 1.2)	1.1 (1.0, 1.1)	1.4 (1.3, 1.4)
Fruit	1.0 (0.9; 1.0)	1.0 (0.9; 1.1)	1.0 (0.9; 1.0)	0.9 (0.8; 1.0)	0.7 (0.6; 0.8)	0.9 (0.9; 1.0)	1.3 (1.2; 1.3)	1.0 (0.9; 1.1)	1.0 (0.9; 1.1)	1.0 (0.9; 1.0)	0.8 (0.7, 0.8)	1.1 (1.1, 1.2)
Wholemeal cereals	0.9 (0.8; 1.0)	1.3 (1.2; 1.4)	0.9 (0.7; 1.0)	0.5 (0.4; 0.6)	0.5 (0.4; 0.6)	0.9 (0.8; 1.0)	1.3 (1.1; 1.4)	0.9 (0.8; 1.0)	1.0 (0.8; 1.1)	0.8 (0.7; 0.9)	0.6 (0.5, 0.7)	1.1 (1.0, 1.2)
Potatoes	0.6 (0.6; 0.7)	0.4 (0.3; 0.4)	0.7 (0.6; 0.7)	0.8 (0.8; 0.8)	0.7 (0.6; 0.7)	0.6 (0.5; 0.6)	0.6 (0.6; 0.7)	0.7 (0.6; 0.7)	0.6 (0.5; 0.6)	0.6 (0.6; 0.7)	0.7 (0.6, 0.7)	0.6 (0.5, 0.6)
Vegetable oils	0.6 (0.6; 0.6)	0.5 (0.5; 0.6)	0.6 (0.6; 0.7)	0.7 (0.7; 0.8)	0.5 (0.4; 0.5)	0.7 (0.6; 0.7)	0.7 (0.7; 0.8)	0.6 (0.6; 0.7)	0.6 (0.5; 0.6)	0.6 (0.6; 0.7)	0.6 (0.5, 0.6)	0.7 (0.6, 0.7)
Other fats	0.5 (0.4; 0.5)	0.3 (0.2; 0.4)	0.5 (0.4; 0.6)	0.6 (0.5; 0.7)	0.6 (0.5; 0.7)	0.3 (0.2; 0.4)	0.5 (0.4; 0.6)	0.1 (0.1; 0.1)	0.2 (0.1; 0.2)	1.1 (1.0; 1.2)	0.5 (0.4, 0.6)	0.4 (0.4, 0.5)
Cheese	0.4 (0.3; 0.4)	0.3 (0.3; 0.4)	0.4 (0.3; 0.4)	0.4 (0.4; 0.5)	0.3 (0.2; 0.3)	0.3 (0.3; 0.4)	0.5 (0.4; 0.5)	0.4 (0.3; 0.4)	0.3 (0.3; 0.4)	0.4 (0.4; 0.5)	0.4 (0.3, 0.4)	0.4 (0.3, 0.4)
White meat	0.4 (0.4; 0.5)	0.4 (0.3; 0.4)	0.5 (0.4; 0.5)	0.5 (0.5; 0.5)	0.4 (0.4; 0.5)	0.4 (0.4; 0.5)	0.5 (0.4; 0.5)	0.4 (0.3; 0.4)	0.5 (0.4; 0.5)	0.5 (0.5; 0.6)	0.4 (0.4, 0.5)	0.4 (0.4, 0.5)
Juices	0.5 (0.4; 0.5)	0.3 (0.3; 0.4)	0.5 (0.4; 0.6)	0.6 (0.5; 0.7)	0.2 (0.2; 0.3)	0.4 (0.3; 0.5)	0.8 (0.7; 0.9)	0.5 (0.4; 0.6)	0.5 (0.4; 0.6)	0.4 (0.4; 0.5)	0.4 (0.3, 0.4)	0.6 (0.5, 0.7)
Eggs	0.4 (0.3; 0.4)	0.3 (0.3; 0.3)	0.3 (0.3; 0.4)	0.4 (0.4; 0.5)	0.2 (0.2; 0.3)	0.3 (0.3; 0.4)	0.5 (0.5; 0.6)	0.4 (0.4; 0.5)	0.3 (0.3; 0.4)	0.3 (0.3; 0.4)	0.3 (0.3, 0.4)	0.4 (0.3, 0.4)

Sweetened milk beverages and flavored cheese curds	0.3 (0.3; 0.4)	0.2 (0.1; 0.2)	0.3 (0.2; 0.4)	0.5 (0.4; 0.6)	0.2 (0.1; 0.2)	0.3 (0.2; 0.4)	0.5 (0.4; 0.6)	0.2 (0.1; 0.2)	0.3 (0.2; 0.4)	0.5 (0.4; 0.6)	0.4 (0.3, 0.4)	0.3 (0.2, 0.3)
Fish	0.3 (0.2; 0.3)	0.3 (0.2; 0.4)	0.3 (0.2; 0.3)	0.3 (0.2; 0.3)	0.1 (0.1; 0.2)	0.2 (0.2; 0.3)	0.5 (0.4; 0.5)	0.2 (0.2; 0.3)	0.3 (0.2; 0.3)	0.3 (0.2; 0.3)	0.2 (0.2, 0.2)	0.3 (0.3, 0.4)
Nuts and seeds	0.3 (0.3; 0.4)	0.5 (0.4; 0.6)	0.3 (0.2; 0.3)	0.2 (0.1; 0.2)	0.1 (0.1; 0.2)	0.3 (0.2; 0.4)	0.6 (0.5; 0.7)	0.4 (0.3; 0.5)	0.4 (0.3; 0.5)	0.3 (0.2; 0.3)	0.1 (0.1, 0.1)	0.5 (0.4, 0.6)
Legumes	0.2 (0.2; 0.2)	0.2 (0.2; 0.3)	0.2 (0.2; 0.2)	0.2 (0.2; 0.3)	0.1 (0.1; 0.1)	0.2 (0.1; 0.2)	0.4 (0.3; 0.4)	0.2 (0.1; 0.2)	0.2 (0.2; 0.3)	0.3 (0.2; 0.3)	0.1 (0.1, 0.1)	0.3 (0.3, 0.3)
Breakfast cereals	0.2 (0.1; 0.2)	0.1 (0.1; 0.2)	0.2 (0.1; 0.2)	0.2 (0.1; 0.2)	0.0 (0.0; 0.1)	0.2 (0.1; 0.2)	0.3 (0.2; 0.3)	0.1 (0.0; 0.1)	0.2 (0.1; 0.2)	0.3 (0.2; 0.3)	0.1 (0.1, 0.2)	0.2 (0.2, 0.2)
Sweetened beverages and energy drinks	0.1 (0.0; 0.1)	0.0 (0.0; 0.0)	0.0 (0.0; 0.0)	0.1 (0.1; 0.2)	0.0 (0.0; 0.1)	0.0 (0.0; 0.1)	0.1 (0.1; 0.1)	0.0 (0.0; 0.1)	0.0 (0.0; 0.1)	0.1 (0.1; 0.1)	0.0 (0.0, 0.1)	0.1 (0.0, 0.1)
**Food consumption (g)**[$]												
Cold cereal	7.8 (6.6, 9.1)	10. 0 (7.5, 12.6)	7.8 (5.6, 9.9)	5.7 (3.9, 7.4)	3.3 (1.9, 4.7)	8.4 (6.1, 10.7)	11.8 (9.3, 14.2)	4.3 (2.7, 5.9)	8.3 (6.1, 10.5)	10.8 (8.3, 13.3)	5.0 (3.5, 6.4)	10.1 (8.3, 12.0)
Skim milk, on cereal or to drink	10.9 (7.0, 14.7)	18.0 (9.9, 26.1)	8.4 (0.9, 15.9)	6.3 (3.0, 9.7)	4.8 (2.2, 11.8)	6.7 (3.3, 10.1)	21.0 (12.7, 29.3)	4.9 (1.5, 8.2)	11.0 (5.0, 16.9)	16.8 (7.5, 26.0)	5.8 (0.4, 11.2)	15.0 (9.7, 20.4)
Orange juice or grapefruit juice	61.6 (53.2, 69.9)	41.9 (30.4, 53.3)	57.7 (43.9, 71.6)	85.1 (68.1, 102.1)	32.6 (22.8, 42.5)	54.5 (41.2, 67.7)	97.2 (79.8, 114.6)	64.1 (49.0, 79.1)	63.8 (50.1, 77.5)	56.9 (42.0, 71.9)	49.6 (39.0, 60.2)	71.4 (59.0, 83.8)
Fruit (not juices)	116.2 (110.6, 121.7)	121.1 (112.3, 129.8)	114.7 (104.9, 124.5)	112.8 (102.4, 123.2)	85.8 (79.0, 92.5)	113.7 (105.4, 122.1)	148.6 (138.1, 159.1)	117.1 (107.1, 127.1)	118.2 (108.9, 127.6)	113.2 (103.5, 122.8)	95.5 (87.9, 103.1)	133.1 (125.8, 140.4)
Eggs, fried or scrambled in margarine, butter or oil	15.7 (14.2, 17.1)	13.4 (11.2, 15.6)	14.9 (12.3, 17.5)	18.6 (16.0, 21.3)	8.8 (7.2, 10.3)	15.4 (12.8, 18.0)	22.7 (20.0, 25.4)	19.0 (16.0, 21.9)	13.2 (11.1, 15.3)	14.8 (12.4, 17.1)	13.4 (11.6, 15.2)	17.5 (15.3, 19.6)
Sausage or bacon, regular-fat	5.3 (4.6, 6.0)	2.0 (1.4, 2.6)	5.0 (4.0, 5.9)	8.8 (7.3, 10.3)	5.8 (4.5, 7.1)	5.2 (4.2, 6.3)	4.8 (3.7, 6.0)	5.7 (4.4, 7.1)	5.2 (3.9, 6.4)	4.9 (4.0, 5.9)	7.3 (6.1, 8.5)	3.7 (3.0, 4.3)
Beef or pork hot dogs, regular fat	0.8 (0.5, 1.1)	0.3 (0.1, 0.5)	0.7 (0.3, 1.1)	1.5 (0.8, 2.3)	0.5 (0.1, 0.9)	0.7 (0.4, 1.0)	1.3 (0.6, 2.0)	0.4 (0.2, 0.6)	0.6 (0.3, 0.8)	1.5 (0.7, 2.3)	0.8 (0.4, 1.3)	0.8 (0.5, 1.2)
Cheese or cheese spread, regular-fat	8.7 (8.0, 9.5)	8.6 (7.2, 10.1)	8.5 (7.1, 9.9)	9.0 (7.9, 10.2)	6.8 (5.5, 8.0)	8.2 (7.1, 9.3)	11.2 (9.7, 12.6)	7.9 (6.7, 9.1)	8.7 (7.5, 9.9)	9.6 (8.0, 11.1)	8.0 (6.9, 9.2)	9.3 (8.3, 10.3)
French fries, home fries, or hash brown potatoes	2.8 (2.3, 3.4)	1.2 (0.9, 1.6)	2.8 (2.0, 3.6)	4.5 (3.2, 5.8)	2.2 (1.6, 2.8)	2.6 (1.6, 3.5)	3.8 (2.6, 4.9)	2.8 (1.7, 3.9)	2.5 (1.8, 3.3)	3.2 (2.3, 4.1)	3.0 (2.2, 3.9)	2.7 (2.0, 3.4)

Rice	90.0 (84.9, 95.0)	65.3 (56.7, 74.0)	95.4 (87.2, 103,6)	108.9 (100.7, 117.1)	93.7 (84.9, 102.6)	85.7 (76.7, 94.7)	90.5 (81.7, 99.2)	97.3 (89.2, 105.5)	85.4 (76.3, 94.5)	87.1 (77.9, 96.3)	94.8 (87.3, 102.2)	86.0 (79.0, 93.0)
Mayonnaise, regular-fat	0.6 (0.4, 0.7)	0.2 (0.1, 0.3)	0.5 (0.3, 0.6)	1.0 (0.7, 1.2)	0.4 (0.2, 0.5)	0.3 (0.3, 0.4)	1.0 (0.7, 1.3)	0.4 (0.3, 0.5)	0.5 (0.4, 0.7)	0.7 (0.5, 1.0)	0.5 (0.4, 0.7)	0.6 (0.4, 0.8)
Salad dressings, regular-fat	1.8 (1.3, 2.4)	1.4 (0.4, 2.5)	1.5 (0.6, 2.4)	2.6 (1.5, 3.6)	1.2 (0.3, 2.1)	2.3 (1.0, 3.7)	2.0 (1.4, 2.5)	2.2 (0.9, 3.5)	1.3 (0.8, 1.9)	2.0 (1.1, 3.0)	1.9 (1.0, 2.9)	1.8 (1.1, 2.4)
Margarine or butter on bread, rolls, pancakes	6.4 (6.0, 6.8)	4.1 (3.5, 4.6)	6.7 (6.2, 7.3)	8.3 (7.6, 9.0)	6.8 (6.1, 7.4)	6.0 (5.3, 6.6)	6.4 (5.7, 7.1)	7.6 (7.0, 8.3)	4.6 (4.0, 5.3)	6.8 (6.2, 7.5)	7.4 (6.8, 7.9)	5.6 (5.1, 6.1)
Margarine or butter on vegetables, including potatoes	0.4 (0.3, 0.5)	0.1 (0.1, 0.2)	0.4 (0.2, 0.5)	0.6 (0.4, 0.8)	0.2 (0.1, 0.3)	0.5 (0.3, 0.6)	0.5 (0.3, 0.7)	0.4 (0.2, 0.5)	0.4 (0.2, 0.5)	0.4 (0.3, 0.6)	0.3 (0.2, 0.5)	0.4 (0.3, 0.6)
Margarine, butter, or oil on rice or pasta	0.3 (0.2, 0.4)	0.2 (0.0, 0.3)	0.2 (0.1, 0.3)	0.5 (0.3, 0.7)	0.1 (0.0, 0.2)	0.4 (0.2, 0.6)	0.3 (0.2, 0.5)	0.3 (0.1, 0.5)	0.3 (0.1, 0.4)	0.3 (0.2, 0.4)	0.2 (0.1, 0.4)	0.3 (0.2, 0.4)
Percentage energy from dietary fat$	31.3 (31.0, 31.7)	29.8 (29.4, 30.3)	31.2 (30.7, 31.6)	33.0 (32.4, 33.6)	31.6 (31.1, 32.1)	31.4 (30.9, 32.0)	31.0 (30.4, 31.6)	32.1 (31.6, 32.7)	30.6 (30.1, 31.1)	31.3 (30.7, 31.9)	32.4 (31.9, 32.9)	30.5 (30.1, 30.9)
Total fat intake (g)$	7.1 (6.6, 7.5)	4.4 (3.8, 5.0)	7.3 (6.7, 7.9)	9.5 (8.7, 10.2)	7.1 (6.4, 7.7)	6.8 (6.2, 7.5)	7.3 (6.4, 8.1)	8.3 (7.6, 9.0)	5.3 (4.6, 6.0)	7.6 (6.9, 8.2)	7.9 (7.4, 8.5)	6.3 (5.8, 6.9)
Regular fat intake (g)$	6.2 (5.8, 6.6)	3.7 (3.2, 4.3)	6.3 (5.7, 6.9)	8.6 (7.9, 9.3)	6.1 (5.5, 6.8)	6.2 (5.5, 6.8)	6.3 (5.6, 7.1)	8.1 (7.4, 8.7)	4.8 (4.1, 5.4)	5.8 (5.1, 6.4)	7.2 (6.6, 7.8)	5.4 (4.9, 6.0)

'Polish-aMED' – 'Polish-adapted Mediterranean Diet' (range of points: 0–8), levels (in points): 'lower' (0–4), 'higher' (5–8); #the frequency consumption was expressed as a times/day after assigning the values for categories of frequency consumption as follows: 'never or almost never' = 0; 'once a month or less' = 0.025; 'several times a month' = 0.1; 'several times a week' = 0.571; 'daily' = 1; 'several times a day' = 2; $the food consumption was expressed in grams, and the total fat intake, regular fat intake, and the percentage energy from fat were calculated according to the Scoring Procedures of the Percentage Energy from Fat Screener (description in the Material and Methods section); 95%CI – 95% confidence interval.

Table S3. Description of food groups for the 'Polish-adapted Mediterranean Diet' score (0–8 points) calculation – data for the Initial control sample (*n* = 242) [58].

Food groups/food items	Frequency of consumption (times/day)*		Criteria for 1 point
	Mean (95% CI)	Median	
**VEGETABLES**: all kinds of vegetables: cruciferous vegetables (cabbages, brussel sprouts, cauliflower, broccoli, kale, etc.), yellow-orange vegetables (carrots, peppers, etc.), leafy green vegetables (spinach, chicory, lettuce, rocket, leek, celery, parsley, etc.), tomatoes, gourds and squashes (fresh cucumber, marrow, courgettes, pumpkins, aubergines, etc.), root vegetables and other (parsnip, beetroots, onion, garlic, celeriac, radishes, turnip, salads and mixed vegetables, etc.)	1.300 (1.230; 1.371)	1.000	Greater than median intake (times/day)*
**FRUIT**: all kinds of fruit: stone fruit (apricots, cherries, nectarines, peaches, plums, grapes, etc.), kiwi and citrus fruit (kiwi, oranges, mandarins, grapefruit, lemons, pomelos, etc.), tropical fruits (pineapples, watermelon, melons, fresh dates and figs, etc.), berries (strawberries, raspberries, blackberries, blueberries, redcurrants, blackcurrants, etc.), bananas, apples, pears, avocado	0.989 (0.925; 1.052)	1.000	Greater than median intake (times/day)*
**WHOLEMEAL CEREALS**: wholemeal wheat or rye bread, seeded loafs, pumpernickel, wholemeal groats, wholemeal rice, wholemeal pasta	0.890 (0.799; 0.981)	0.671	Greater than median intake (times/day)*
**FISH**: lean fish (ollock, cod, perch, hake, carp to 1 kg, tuna, panga, trout, etc.) oily fish (salmon, sardines, herring, mackerel, eel, large carp, etc.)	0.293 (0.253; 0.334)	0.200	Greater than median intake (times/day)*
**LEGUMES**: fresh and tinned legumes (corn, green peas, green beans, etc.), dry and processed pulses beans (fava, broad, French, green), soya, peas, chickpea and processed pulses (baked beans, hummus, other bread spreads)	0.223 (0.187; 0.259)	0.125	Greater than median intake (times/day)*
**NUTS** and **SEEDS**: peanuts, hazelnuts, walnuts, almonds, pistachios, cashews, coconuts, chestnuts, etc., pumpkin seeds, sesame seeds, sunflower seeds, wheat germs, etc.	0.375 (0.312; 0.438)	0.125	Greater than median intake (times/day)*
**RATIO of VEGETABLES OILS** (rapeseed oil, sunflower oil, linseed oil, olives) **to ANIMAL FAT** (butter, cream, lard) instead of RATIO of MONOUNSATURATED to SATURATED FAT	1.462 (1.125; 1.799)	0.519	Greater than median intake (times/day)*
**RED** and **PROCESED MEAT**: red meat (pork, beef, veal, etc.), sausages, bacon, reconstituted meat (sausages, meat loaf, hot-dogs, smoked sausages, bacon, etc.), high quality cured meats (ham, poultry and pork-beef good quality cold meats, etc.), offal products (liver, blood sausage, sweetbread, liver pate,etc.),game (wild boar, venison, quail, mallard, hare, etc.)	1.252 (1.151; 1.352)	1.267	Lower than median intake (times/day)*

* Food frequency consumption was expressed as a times/day after assigning the values for categories of frequencies as follows: 'never or almost never' = 0; 'once a month or less' = 0.025; 'several times a month' = 0.1; 'several times a week' = 0.571; 'daily' = 1; 'several times a day' = 2; 95% CI – 95% confidence interval.

Table S4. Confounders in the case-control study regarding association of dietary fat intake with dietary patterns and breast cancer occurrence.

Confounders	Categories
**Age** (years)	40.0–49.9; 50.0–59.9; 60.0–69.9; 70.0–79.9
**BMI** (kg/m^2) calculated using measured weight and height	≤24.9; 25.0–29.9; ≥30.0
**Socioeconomic status (SES)** after combining data based on SES' factors:	low; average; high
place of residence	village
	town <20,000 inhabitants
	town 20,000–100,000 inhabitants
	city >100,000 inhabitants
educational level	primary

	secondary	
	higher	
economic situation (self-declared)	below average	
	average	
	above average	
situation of household (self-declared)	we live poorly – I don't have enough resources even for basic needs (food/clothing/housing fees)	
	we live very thriftily – I have enough resources only for basic needs (food/clothing/housing fees)	
	we live thriftily – so I have enough resources for everything	
	we live well – I have enough resources for everything, but I don't put off savings	
	we live very well – I have enough resources for everything and I put off savings	
**Menopausal status**	peri-, postmenopausal	
**Age at menarche** (years)	<12.0; 12–14.9; ≥15.0	
**Oral contraceptive use** (ever)	no; yes	
**Hormone-replacement therapy use**	no; yes	
**Number of full-term pregnancies**	0; 1–2; ≥3	
**Overall physical activity** after combining data based on physical activity at work and physical activity in leisure time	low; moderate; high	
physical activity at work	low	more than 70% of working time spent sedentary or retired
	moderate	approx. 50% of working time spent sedentary and 50% of working time spent in an active manner
	high	approx. 70% of working time spent in an active manner or physical work related to great exertion
physical activity in leisure time	low	sedentary for most of the time, watching TV, reading books, walking 1–2 hours per week
	moderate	walking, bike riding, gymnastics, gardening, light physical activity performed 2–3 hours per week
	high	bike riding, jogging, gardening, sport activities involving physical exertion performed more than 3 hours weekly
**Abuse of alcohol**	no	
	yes	intake at least 1 bottle (0.5 liters) of beer or 2 glasses of wine (300ml) or 2 drinks (300ml) or 2 glasses of vodka (60ml) per day
**Smoking status**	non-smoker	
	smoker	current-smoker or/ and former-smoker
**Vitamin/mineral supplements use** (within last 12 months)	no; yes	
**Family history of breast cancer in first- or second-degree relatives**	no; I don't know; yes	
**Molecular of breast cancer subtypes**	triple negative; ER-, PR-, HER2+ subtype; luminal A; luminal B	

Table S5. The dietary fat intake in association with dietary patterns (% or mean (SD)).

**Variable**	**Total sample**	**Energy from dietary fat (tertiles)**			**p-value**
		**bottom (<30%)**	**middle (30–32%)**	**upper (>32%)**	
Sample size	420	139	141	140	
Energy from dietary fat (%)[#]	31.3 (3.3)	28.1 (1.5)	31.1 (0.8)	34.9 (2.5)	<0.0001
Total fat intake (g)[#]	7.1 (4.3)	4.5 (3.4)	6.8 (3.4)	9.9 (4.3)	<0.0001

<5	35.2	56.1	36.2	13.6	
5–8	31.2	33.1	35.5	25.0	<0.0001
>8	33.6	10.8	28.4	61.4	
Regular fat intake (g)[#]	6.2 (4.2)	3.4 (3.1)	5.9 (3.2)	9.3 (4.0)	<0.0001
<5	33.6	60.4	31.2	9.3	
5–7	33.3	32.4	44.0	23.6	<0.0001
>7	33.1	7.2	24.8	67.1	
Dietary patterns					
'Non-Healthy' score (points)[#]	3.5 (1.8)	2.6 (1.6)	3.4 (1.6)	4.6 (1.7)	<0.0001
tertiles					
bottom	33.1	51.1	35.5	12.9	
middle	33.6	30.9	39.7	30.0	<0.0001
upper	33.3	18.0	24.8	57.1	
'Prudent' score (points)[#]	3.4 (1.2)	3.7 (1.2)	3.3 (1.3)	3.3 (1.2)	0.0086
tertiles					
bottom	33.1	25.9	40.4	32.9	
middle	33.3	34.5	29.1	36.4	0.0945
upper	33.6	39.6	30.5	30.7	
'Margarine and Sweetened Dairy' score (points)[#]	0.1 (1.0)	0.1 (0.7)	0.2 (1.0)	0.0 (1.2)	0.0380
tertiles					
bottom	33.3	23.0	34.8	42.1	
middle	33.1	45.3	29.8	24.3	0.0012
upper	33.6	31.7	35.5	33.6	
Polish-aMED® score (points)[#]	4.7 (1.8)	5.5 (1.5)	4.7 (1.7)	3.9 (1.7)	<0.0001
lower (0–4 points)	45.0	25.2	46.1	63.6	
higher (5–8 points)	55.0	74.8	53.9	36.4	<0.0001

Polish-aMED® – 'Polish-adapted Mediterranean Diet' (range of points: 0–8); the procedure of the total fat intake, regular fat intake and percentage of energy from dietary fat - calculation are given in the Materials and Methods section; % – sample percentage; [#]mean (SD); *p*-value – level of significance was assessed by chi^2 test (categorical variables) or Kruskal-Wallis' test (continuous variables).

Table S6. The consumption of single foods and the assessment of fat content in the diet by the percentage energy from dietary fat status (% or mean (SD)).

**Variable**	**Energy from dietary fat (%)**			***p*-value**
	**<30**	**30–32**	**>32**	
Sample Size	139	141	140	
Food consumption (g)[#]				
Cold cereal	11.8 (15.2)	6.9 (12.4)	4.8 (9.8)	0.0001
Skim milk, on cereal or to drink	19.5 (62.6)	7.8 (22.6)	5.4 (17.6)	0.8024
Orange juice or grapefruit juice	91.0 (102.1)	50.2 (77.0)	43.9 (72.4)	0.0007
Fruit (not juices)	134.9 (60.3)	113.2 (55.9)	100.5 (52.4)	<0.0001
Eggs, fried or scrambled in margarine, butter or oil	13.8 (14.4)	14.3 (13.4)	18.9 (16.8)	0.0051
Sausage or bacon, regular-fat	1.8 (3.1)	4.4 (5.4)	9.6 (8.8)	<0.0001
Beef or pork hot dogs, regular fat	0.2 (0.8)	0.6 (1.6)	1.7 (4.8)	0.0007
Cheese or cheese spread, regular-fat	6.1 (6.3)	9.0 (7.1)	11.1 (9.2)	<0.0001
French fries, home fries, or hash brown potatoes	1.9 (3.8)	2.2 (3.6)	4.5 (7.7)	0.0008
Rice	99.4 (52.9)	85.0 (51.6)	85.5 (53.7)	0.0099
Mayonnaise, regular-fat	0.3 (0.6)	0.4 (0.6)	1.0 (1.7)	<0.0001
Salad dressings, regular-fat	0.5 (1.1)	1.0 (1.7)	4.0 (9.8)	<0.0001
Margarine or butter on bread, rolls, pancakes	4.2 (3.3)	6.2 (3.3)	8.7 (4.0)	<0.0001
Margarine or butter on vegetables, including potatoes	0.1 (0.5)	0.3 (0.8)	0.7 (1.2)	<0.0001
Margarine, butter, or oil on rice or pasta	0.1 (0.7)	0.2 (0.6)	0.5 (1.4)	0.0008
Frequency of reduced-fat margarine use				
didn't use/almost never	71.2	78.0	85.7	
about 1/4 of the time	6.5	11.3	7.1	
about 1/2 of the time	11.5	6.4	5.7	0.0060

about 3/4 of the time	6.5	3.5	1.4	
almost always or always	4.3	0.7	0.0	
Fat content in the diet (self-declared)				
low	28.8	20.6	15.7	
medium	67.6	63.8	65.7	0.0006
high	3.6	15.6	18.6	
Comparison of fat content in the diet (objective vs subjective assessment)				
low vs low				
low vs average	28.8	3.5	0.0	
low vs high	0.0	17.0	15.7	
average vs average	67.6	7.1	0.0	
average vs low				<0.0001
average vs high	0.0	56.7	65.7	
high vs high	0.0	14.9	18.6	
high vs low				
high vs average	3.6	0.7	0.0	
Consistent assessment of comparison of fat content in the diet (objective vs subjective)	67.6	22.0	18.6	<0.0001

% – sample percentage; #mean (SD); *p*-value – level of significance assessed by chi^2 test (categorical variables) or Kruskal-Wallis' test (continuous variables).

Table S7. The consumption of single foods and the assessment of fat content in the diet by the by case-control status (% or mean (SD)).

Variable	Cancer-Control Sample	Cancer Sample	Control Sample	*p*-Value
Sample Size	420	190	230	
Food consumption (g)#				
Cold cereal	7.8 (13.0)	5.8 (10.9)	9.5 (14.3)	0.0006
Skim milk, on cereal or to drink	10.9 (40.0)	13.7 (47.0)	8.6 (33.1)	0.0098
Orange juice or grapefruit juice	61.6 (87.1)	66.7 (91.9)	57.3 (82.9)	0.9011
Fruit (not juices)	116.2 (57.9)	113.4 (58.7)	118.4 (57.3)	0.1166
Eggs, fried or scrambled in margarine, butter or oil	15.7 (15.1)	17.4 (16.0)	14.2 (14.2)	0.0743
Sausage or bacon, regular-fat	5.3 (7.0)	5.6 (6.8)	5.0 (7.2)	0.0341
Beef or pork hot dogs, regular fat	0.8 (3.0)	1.3 (4.0)	0.4 (1.9)	0.0021
Cheese or cheese spread, regular-fat	8.7 (7.9)	8.1 (6.6)	9.2 (8.8)	0.7077
French fries, home fries, or hash brown potatoes	2.8 (5.5)	3.5 (6.3)	2.3 (4.7)	0.0031
Rice	90.0 (53.0)	94.0 (52.9)	86.6 (53.1)	0.0741
Mayonnaise, regular-fat	0.6 (1.1)	0.7 (1.3)	0.5 (0.9)	0.1018
Salad dressings, regular-fat	1.8 (5.9)	2.8 (8.3)	1.1 (2.6)	0.0584
Margarine or butter on bread, rolls, pancakes	6.4 (4.0)	7.0 (4.3)	5.8 (3.6)	0.0046
Margarine or butter on vegetables, including potatoes	0.4 (0.9)	0.5 (1.0)	0.3 (0.8)	0.2876
Margarine, butter, or oil on rice or pasta	0.3 (1.0)	0.4 (1.0)	0.2 (0.9)	0.0170
Frequency of reduced-fat margarine use				
didn't use/almost never	78.3	73.7	82.2	
about 1/4 of the time	8.3	11.6	5.7	
about 1/2 of the time	7.9	10.0	6.1	0.0602
about 3/4 of the time	3.8	2.6	4.8	
almost always or always	1.7	2.1	1.3	
Fat content in the diet (self-declared)				
low	21.7	14.7	27.4	
medium	65.7	67.4	64.3	<0.001
high	12.6	17.9	8.3	
Comparison of fat content in the diet (objective vs subjective assessment)				
low vs low				
low vs average	10.7	6.8	13.9	
low vs high	11.0	7.9	13.5	
average vs average	24.8	20.5	28.3	
average vs low				<0.001

average vs high	41.0	46.8	36.1	
high vs high	11.2	15.8	7.4	
high vs low				
high vs average	1.4	2.1	0.9	
Consistent assessment of comparison of fat content in the diet (objective vs subjective)	36.0	36.3	35.7	0.8878

% – sample percentage; #mean (SD); *p*-value – level of significance assessed by chi^2 test (categorical variables) or Kruskal-Wallis' test (continuous variables).
